# Stereotypical conceptions of women’s roles in contemporary Kyrgyz society: a psychological analysis

**DOI:** 10.3389/fpsyg.2026.1857963

**Published:** 2026-08-05

**Authors:** Kamilla Turdubaeva, Asel Moldosh

**Affiliations:** Department of Psychology, Kyrgyz National University named after Jusup Balasagyn, Bishkek, Kyrgyzstan

**Keywords:** ambivalent gender attitudes, Central Asia, family relations, gender stereotypes, gender-role traditionalism, Kyrgyz Republic, women’s roles

## Abstract

**Introduction:**

Women’s social roles in the Kyrgyz Republic are shaped by the coexistence of long-established family-centered expectations and expanding participation in education, employment, and public life. This study examined perceived media images of women, benevolent and hostile sexism, gender-role traditionalism, and cultural-value orientations, and identified the attitudinal, cultural, and demographic correlates of gender-role traditionalism.

**Methods:**

A quantitative cross-sectional online survey was conducted with 242 adults residing in the Kyrgyz Republic, recruited through non-probability convenience and network-based sampling. Participants completed an author-designed questionnaire, the 12-item Ambivalent Sexism Inventory, the 10-item Gender Role Beliefs Scale, and L. G. Pochebut’s Cultural-Value Orientations Questionnaire. Data were analyzed using paired-samples t-tests, correlations, and hierarchical multiple regression.

**Results:**

The most frequently perceived media image was the “mother and guardian of the hearth” (74.0%), followed by the obedient daughter and wife who follows tradition (48.8%). Benevolent sexism (M = 3.01, SD = 1.14) was endorsed more strongly than hostile sexism (M = 1.92, SD = 1.07), *t* (241) = 14.65, *p* < 0.001, *d_z* = 0.94. Across the five cultural-value items, responses corresponding to Pochebut’s modern-culture type were most frequent (48.2% of all selections), whereas traditional-culture and dynamically developing-culture responses were similar in frequency (25.9 and 26.0%, respectively). On the social-relations item, 39.3% selected Option B (the collectivist/collateral extended-family response), which was weakly associated with gender-role traditionalism (*r* = 0.16). In the primary hierarchical regression, hostile sexism (*β* = 0.30), benevolent sexism (*β* = 0.26), and the Option B social-relations response (*β* = 0.13) were significant predictors of traditionalism after adjustment for age, gender, education, and residence. However, a three-category robustness check showed that the social-relations item did not explain significant incremental variance beyond the demographic and sexism-related predictors. Education and urban or rural residence were not significantly associated with traditionalism in the full model.

**Discussion:**

Benevolent and hostile sexism remained the most robust predictors of gender-role traditionalism across model specifications. The findings suggest that family-centered role representations remain salient in this non-probability sample and that traditional gender-role beliefs are systematically related to both protective-paternalistic and hostile attitudinal dimensions. The study contributes quantitative evidence on role expectations in a post-Soviet Central Asian context and supports the need for culturally grounded psychological research.

## 1 Introduction

Gender stereotypes are socially shared cognitive structures that organize expectations about the attributes, behaviors, and social positions considered appropriate for women and men. They comprise descriptive beliefs about how members of each gender are presumed to behave and prescriptive beliefs about how they ought to behave. As enduring mental schemas, stereotypes simplify the processing of social information and facilitate interpersonal categorization; at the same time, they may also constrain individual choices, shape evaluations of competence and suitability, and, according to social-psychological accounts, help legitimize prevailing distributions of social roles and authority (Bai et al., 2024; Ellemers, 2018). Contemporary evidence indicates that gender stereotypes continue to influence how women are evaluated in occupational and leadership contexts even where their formal participation in education and employment has expanded (Heilman et al., 2024).

Gender stereotypes are shaped by cultural norms, accumulated collective experience, institutional arrangements, and the historically established distribution of women and men across family, occupational, and public roles. Through socialization, these shared representations become relevant not only to how individuals perceive others but also to how they understand and evaluate themselves, influencing self-concept, occupational aspirations, interpersonal behavior, family decision-making, and perceptions of socially appropriate femininity and masculinity (Bem, 1981; Eagly and Wood, 2012). Understanding how such expectations are formed, transmitted, and internalized is therefore central to social and cultural psychology, particularly in societies undergoing rapid economic and cultural transformation.

Gender stereotypes are neither entirely stable nor uniformly disappearing. Cross-temporal evidence suggests that perceptions of women’s competence have become more favorable in some societies, partly reflecting women’s increased participation in education and paid employment. Nevertheless, communal qualities such as warmth, care, emotional sensitivity, and family orientation continue to be associated more strongly with women, whereas agency, independence, assertiveness, and authority remain more closely associated with men (Eagly et al., 2020). Stereotype content also varies according to social position, occupation, family role, and age (Bye et al., 2022). Structural changes in women’s participation therefore do not necessarily produce immediate or equivalent changes in culturally embedded expectations.

These processes are particularly salient in societies where traditional family arrangements coexist with expanding educational, economic, and professional opportunities for women. In such contexts, women may increasingly be recognized as professionals, economic providers, and public figures while continuing to be evaluated primarily through expectations related to motherhood, domestic responsibility, modesty, and family harmony. Traditional and contemporary images of womanhood may thus coexist within the same cultural system rather than simply replacing one another, with different expectations becoming salient in different settings (Eagly et al., 2020; Heilman et al., 2024).

The Kyrgyz Republic provides a theoretically informative context for examining this coexistence. Gender-role expectations in Kyrgyzstan have been shaped by the interaction of nomadic heritage, kinship organization, Soviet modernization, post-Soviet economic restructuring, religious and cultural traditions, urbanization, migration, and globally circulating models of women’s education and public participation (Botokanova, 2022). These influences have not unfolded as a linear process in which contemporary roles replace traditional ones; instead, they intersect within present-day social life, allowing family-centered and achievement-oriented expectations to remain simultaneously meaningful. Kyrgyz society is further characterized by strong family interdependence, kinship obligations, and concern with collective reputation, expressed through cultural concepts such as *Uyat* (shame) and *Namys* (honor) (Botokanova, 2022; Sydykova, 2019). Although these concepts can support dignity, responsibility, and solidarity, their gendered application may also generate differentiated behavioral expectations for women and men. The present study therefore approaches gender stereotypes not merely as individual prejudices but as cognitive and normative representations situated within a broader relational and cultural system.

To provide an explicitly psychological account of these processes, the study is grounded primarily in Social Role Theory and Ambivalent Sexism Theory. Gender Schema Theory, Social Identity Theory, System Justification Theory, and Role Strain Theory serve as complementary perspectives that clarify how culturally available expectations may become cognitively internalized, socially reinforced, and connected with competing role demands. These frameworks provide an interpretive lens; they do not imply that every construct associated with them was directly measured in the present research.

### 1.1 Social role theory and the internalization of gender stereotypes

Social Role Theory proposes that gender stereotypes arise partly from the observed distribution of women and men across different social roles (Eagly and Wood, 2012). When women are disproportionately represented in caregiving, domestic, and supportive positions, observers may infer that women inherently possess the communal qualities these roles require—warmth, nurturance, patience, and self-sacrifice—whereas men’s greater representation in leadership and income-generating positions reinforces perceptions of men as agentic, assertive, and suited to authority. Such inferences can consolidate into both descriptive and prescriptive stereotypes: women may not only be perceived as more caring but also expected to prioritize family needs, assume primary responsibility for domestic work, and maintain interpersonal harmony. Individuals who depart from these expectations may be evaluated as socially inappropriate even when their behavior is effective or personally meaningful.

Social Role Theory thus links structural arrangements with individual cognition. Repeated exposure to role-congruent behavior through family relationships, education, media, and everyday interaction makes particular associations between gender and social function cognitively accessible. Through differential socialization, reinforcement, and social sanction, these expectations may become incorporated into self-definition, so that conformity is experienced not solely as a response to external pressure but as a personally meaningful expression of identity.

This process is relevant to the culturally salient Kyrgyz representation of woman as *Ene* (mother) *and* “*Ayal—uydun kutu”* (“A woman is the blessing of the home”)—commonly conveyed through the image of the “mother and guardian of the hearth” (Sydykova, 2019). This image may communicate affection, moral recognition, and respect for motherhood while simultaneously constituting an organized set of expectations concerning caregiving, domestic responsibility, and the maintenance of family cohesion. Its psychological significance lies in its dual operation as a valued cultural identity and a prescriptive standard against which women’s conduct is evaluated.

Social Role Theory does not assume that gender-role expectations are immutable. As women become more visible in education, entrepreneurship, professional employment, and leadership, associated stereotypes may gradually change. However, structural and cognitive change need not occur at the same rate: family-centered expectations may continue to be reproduced through cultural narratives and interpersonal practices even as women’s economic participation expands. Consistent with this, cross-temporal analysis shows that although women’s perceived competence has risen, communal stereotypes have remained stable (Eagly et al., 2020). The simultaneous visibility of the “mother and guardian of the hearth” and the “successful and independent businesswoman” may therefore reflect not the disappearance of traditional beliefs but the coexistence of several models of socially acceptable womanhood—a proposition directly relevant to the present study.

### 1.2 Ambivalent sexism theory

Whereas Social Role Theory explains how the distribution of social roles contributes to the content of gender stereotypes, Ambivalent Sexism Theory describes the evaluative structure through which, according to the theory, traditional gender-role arrangements may be maintained (Glick and Fiske, 1996, 2012). The theory distinguishes between hostile sexism and benevolent sexism as related but conceptually distinct dimensions of gender ideology.

Hostile sexism refers to overtly negative or antagonistic attitudes toward women, particularly those perceived as challenging male authority or seeking to alter established gender relations; it may include beliefs that women exaggerate discrimination or use social influence to gain advantages, and it can operate as a mechanism for sanctioning women who depart from conventional roles. Benevolent sexism consists of subjectively positive but paternalistic beliefs that idealize women as morally refined, nurturing, and deserving of male protection. Although experienced as admiration or courtesy, benevolent sexism is typically conditional on women’s conformity to traditional femininity and portrays women as dependent or especially suited to supportive, family-oriented roles. Within ambivalent sexism theory, benevolent sexism is understood not to devalue women directly but to assign positive characteristics in ways that can accompany differentiated authority and more limited autonomy.

Within this framework, hostile and benevolent sexism are viewed not as opposite positions but as complementary components of a single attitudinal system: hostile content is theorized to sanction departures from conventional roles, whereas benevolent content is theorized to reward conformity to expectations of modesty, loyalty, and family devotion. A recent systematic review concluded that hostile sexism is primarily connected with the protection of male power, whereas benevolent sexism supports traditional gender differentiation through protective paternalism and the idealization of conventionally feminine behavior (Bareket and Fiske, 2023).

This distinction is especially relevant in cultural settings where protection, family cohesion, and complementary responsibilities are positively valued. Statements suggesting that women deserve protection or should be relieved of demanding responsibilities may be interpreted as care rather than discrimination, yet may coexist with reduced recognition of women’s professional authority and autonomy. In the Kyrgyz context, benevolent sexism may be particularly salient because its language overlaps with culturally valued ideals of respect, family responsibility, and protection encoded in *Uyat* and *Namys*. The theoretically relevant question is not whether care is expressed, but whether it is conditional on role conformity and accompanied by assumptions of dependency. This framework yields a direct empirical expectation: in a context where family-centered femininity and protective attitudes are culturally valued, benevolent sexism may receive greater endorsement than hostile sexism. It should be emphasized that hostile and benevolent sexism are technical constructs defined within this research tradition; their use in the present study serves to describe patterns of attitudes and does not constitute a normative evaluation of care, protection, respect, or family commitment as cultural values. Together, Social Role Theory and Ambivalent Sexism Theory constitute the principal theoretical architecture of the present study.

### 1.3 Cognitive, identity-based, and system-justifying mechanisms of stereotype maintenance

Gender Schema Theory specifies the cognitive processes through which socially available gender norms may become internalized and reproduced (Bem, 1981, 1993). Individuals develop associative networks that organize information around culturally meaningful distinctions between femininity and masculinity, influencing attention, memory, interpretation, and self-evaluation by making gender-consistent information especially accessible. Once established, such schemas operate with limited conscious deliberation: new information is assimilated to pre-existing expectations, and individuals may evaluate their own adequacy against culturally defined standards of femininity and masculinity. Because schemas are cognitively economical, they can be resistant to disconfirmation. Women’s increasing participation in education and employment may thus be incorporated into existing schemas without transforming the expectation that women remain primarily responsible for family care—professional activity being accepted while treated as secondary to domestic obligations.

From a social-cognitive perspective, stereotype internalization shapes self-concept through reflected appraisals and autobiographical reasoning (Cadinu et al., 2012; Tomasetto et al., 2011; Sinclair and Carlsson, 2013). When internalized family-centered schemas conflict with professional or educational aspirations, cognitive dissonance may arise as individuals experience tension between personally valued goals and culturally prescribed role expectations (Festinger, 1957; Major and O'Brien, 2005). Although the present study does not measure self-concept, stereotype internalization, or cognitive dissonance directly, these mechanisms provide a theoretically coherent account of how cultural norms become personally consequential and why gender-role expectations may persist despite structural change.

Social Identity Theory adds a relational and group-based dimension to this account (Tajfel and Turner, 1986). Individuals derive part of their self-definition from membership in valued groups, including family, gender, ethnic, and regional communities, and conformity to group expectations may signal loyalty and moral reliability. In interdependent environments, deviation from gender-role expectations may be evaluated not only as an individual choice but as behavior affecting family reputation and collective continuity. The concepts of *Uyat* and *Namys* connect personal conduct with collective moral evaluation and, when applied differentially to women and men, may reinforce gender-specific expectations concerning modesty, mobility, and family responsibility.

System Justification Theory further explains why individuals may defend established arrangements even when these distribute authority unequally (Jost and Kay, 2005). Complementary gender stereotypes can present existing divisions as balanced and mutually beneficial—men as stronger and suited to authority, women as morally superior or more deserving of protection—thereby, according to this account, increasing the perceived legitimacy of prevailing arrangements and rendering existing differences in power and autonomy less salient. This perspective is directly relevant to the possibility that women themselves endorse benevolent sexism or traditional beliefs; from this perspective, such endorsement need not be read as evidence either for or against the fairness of existing arrangements; rather, it indicates that gender-role attitudes are embedded within broader systems of identity, belonging, interdependence, and moral meaning that render them personally meaningful to those who hold them.

These patterns are further illuminated by gender-ideology research, which examines how prescriptive beliefs about men’s and women’s roles operate as system-justifying mechanisms that legitimize existing social arrangements (Kite et al., 2008). Gender ideology encompasses both attitudes toward appropriate role behavior and the normative assumptions that embed these attitudes in institutional and family practices. In the Kyrgyz context, gender ideology may be expressed not only through overt restrictions but also through the idealization of family-centered femininity as a cultural virtue.

Finally, gendered schemas and cultural norms may become consequential when individuals occupy several roles with competing demands. Role Strain Theory proposes that strain arises when obligations exceed available time, energy, or social resources (Goode, 1960), and work–family conflict has been conceptualized as inter-role conflict in which the requirements of the work and family domains become difficult to reconcile (Netemeyer et al., 1996).

This is relevant to women’s changing position in Kyrgyzstan, where economic restructuring, urbanization, and migration have increased women’s participation in paid work without necessarily reducing expectations concerning domestic labor and caregiving; professional participation may thus be added as an extra responsibility rather than serving as a basis for redistributing existing obligations. Importantly, the present study did not directly measure role strain, work–family conflict, cognitive dissonance, self-efficacy, or psychological well-being. These constructs are used only as complementary interpretive perspectives; the findings cannot establish that participants personally experienced role conflict or distress, which would require dedicated instruments and, ideally, longitudinal or qualitative evidence.

### 1.4 Historical and sociocultural context of women’s roles in Kyrgyzstan

The psychological mechanisms outlined above operate within a historically specific cultural context. Historical accounts indicate that women in nomadic Kyrgyz communities occupied a complex position: they contributed substantially to domestic production, household management, caregiving, and kinship relations, and—compared with women in some sedentary Central Asian societies—could enjoy greater mobility and public visibility, without being uniformly subject to seclusion or veiling. This relative mobility nonetheless coexisted with patriarchal authority, a gendered division of labor, and expectations of obedience, modesty, and domestic responsibility (Abramzon, 1990; Botokanova, 2022).

Cultural narratives reinforced the symbolic representation of women as bearers of family unity and intergenerational continuity (Aitmatov and Shakhanov, 1996), although Kyrgyz literature also contains more complex portrayals in which traditional prescriptions coexist with female autonomy and agency (Karaeva and Edilova, 2022; Sydykova, 2019). Kyrgyz constructions of femininity therefore cannot be reduced to a single model of passive subordination.

The Soviet period expanded women’s formal access to education, professional training, and public institutions, yet formal equality did not eliminate gender differentiation within the family: increased employment frequently coexisted with continuing responsibility for domestic labor and childcare, producing the phenomenon commonly described as the double burden (Bekturganova, 2009). Post-Soviet restructuring added further complexity. Declining social protections and expanding migration increased the economic importance of women’s paid work, while national, religious, and cultural revival re-emphasized family-centered identities. Extended-family structures, patrilocal residence, and obligations toward the *Uruu* (wider kin group) continue to provide material support, shared childcare, and security while also exposing personal decisions to collective evaluation. Consistent with this, comparative analysis of Kyrgyzstan and Kazakhstan indicates that patrilocal norms and gendered expectations are associated with women expending greater domestic and productive labor effort amid comparatively limited career prospects, contributing to labor-market inequality (Kovaleva et al., 2025).

Labor migration has intensified these dynamics. Women constituted approximately 40% of labor migrants from the Kyrgyz Republic in recent estimates, a shift that increases women’s economic responsibility and can alter household decision-making (International Organization for Migration, 2025). However, economic change is not always matched by changes in moral expectation. Research in rural Kyrgyzstan found that women’s financial contributions through migration were valued even as departures from traditional child-rearing and marital roles attracted social criticism (Scott et al., 2024), and studies of service provision have documented strong expectations that women preserve marriage and maintain family reputation under adverse circumstances. These findings illustrate the coexistence of traditional and contemporary expectations rather than a straightforward transition between them.

Urbanization, higher education, and digital media may diversify the models of womanhood available to individuals, broadening occupational aspirations and increasing contact with egalitarian discourses. Neither urban residence nor education, however, should be treated as automatically dissolving traditional beliefs, which may remain meaningful across residential and educational groups. Comparative research indicates that gender-role stereotypes concerning leadership and family responsibility remain comparatively pronounced in several Central Asian settings, with substantial within- and between-country variation (Yerimpasheva et al., 2023), and studies in Kyrgyzstan document the continuing relevance of gender stereotypes for educational and occupational choices, participation in science and sport, and leadership perceptions (Seitalieva, 2024; Shabdan et al., 2025).

An intersectional perspective cautions against treating “Kyrgyz women” or “Kyrgyz society” as homogeneous categories. Gender-role expectations may vary according to age, education, place of residence, ethnicity, socioeconomic position, marital and parental status, religiosity, language, and migration experience. The present study considers several demographic characteristics, including gender, age, education, and urban or rural residence, but it does not constitute a comprehensive intersectional analysis. Its findings must therefore be interpreted within the characteristics and limitations of the participating sample.

### 1.5 Research gap, aim, and hypotheses

Existing scholarship offers valuable historical, literary, sociological, and qualitative analyses of women’s roles in Kyrgyzstan, and official reports document gender differences in employment, unpaid care, and public participation (National Statistical Committee of the Kyrgyz Republic, 2024, n.d.). However, comparatively little quantitative social-psychological research has examined culturally salient images of women, ambivalent sexism, gender-role beliefs, and cultural-value orientations within a single empirical design, and the relative prominence of benevolent versus hostile sexism in a post-Soviet Central Asian sample has rarely been assessed with standardized psychometric measures. Several questions therefore remain insufficiently explored: whether benevolent sexism is more strongly endorsed than hostile sexism among adults in Kyrgyzstan; how benevolent sexism, cultural-value orientations, and traditional gender-role beliefs are interrelated; how education and urban or rural residence relate to gender-role traditionalism; and how dominant media images of women correspond to standardized attitudinal measures.

The present study addresses this gap through a quantitative cross-sectional survey of adults recruited from urban and rural areas of the Kyrgyz Republic. It integrates Social Role Theory and Ambivalent Sexism Theory as its principal explanatory frameworks, with Gender Schema Theory, Social Identity Theory, System Justification Theory, and Role Strain Theory as complementary perspectives. The aim of the study was to examine the structure and correlates of stereotypical conceptions of women’s roles among the participating adults, with particular attention to dominant images of women, hostile and benevolent sexism, gender-role beliefs, cultural-value orientations, education, and place of residence. Accordingly, the study pursued four objectives:

1 To systematize the social-psychological and cultural foundations of gender-role stereotypes in the Kyrgyz context;
2 To assess the endorsement of traditional and contemporary images of women, hostile and benevolent sexism, and gender-role beliefs in the study sample;
3 To examine the associations among gender-role beliefs, ambivalent sexism, cultural-value orientations, education, and urban or rural residence;
4 To determine which measured attitudinal, cultural, and demographic variables statistically predict gender-role traditionalism.

Drawing on the theoretical perspectives outlined above, the study advanced four theoretically derived hypotheses:

*H1.* Benevolent sexism will be endorsed more strongly than hostile sexism. Because benevolent sexism is expressed through culturally valued ideals of protection, family responsibility, and respect, it is expected to be more socially acceptable, and therefore more strongly endorsed, than the overtly antagonistic content of hostile sexism.

*H2.* Both benevolent and hostile sexism will be positively associated with gender-role traditionalism, consistent with the view that the two ideologies function as complementary supports for a traditional gender system.

*H3.* The “Relationships” item of Pochebut’s Cultural-Value Orientations Questionnaire will be associated with gender-role traditionalism. In the primary binary specification, Option B on this item—the collectivist (collateral) extended-family response in Pochebut’s “Relationships” item—is expected to show a positive association with traditionalism. However, this association is interpreted as an exploratory item-level finding rather than as evidence for a multidimensional cultural-value orientation.

*H4.* Ambivalent sexism (both benevolent and hostile) and the item-level social-relations indicator will jointly predict gender-role traditionalism after adjustment for demographic characteristics, indicating that attitudinal variables and exploratory cultural-value indicators account for variance in traditionalism beyond sociodemographic position.

In addition to these confirmatory hypotheses, the study addressed an exploratory research question concerning the demographic correlates of gender-role traditionalism: *RQ*. How do age, education, place of residence, and gender relate to gender-role traditionalism and to hostile and benevolent sexism? Because prior evidence on these associations in the Kyrgyz context is limited and, in some cases, mixed, these relationships were examined without directional predictions.

## 2 Materials and methods

### 2.1 Research design and procedure

The study employed a quantitative, cross-sectional online survey design (Creswell and Creswell, 2018) to examine stereotypical conceptions of women’s roles and their associations with ambivalent sexism, gender-role beliefs, cultural-value orientations, and sociodemographic characteristics among adults residing in the Kyrgyz Republic. Because the study used an observational design without manipulation of variables or assignment to experimental conditions, the findings were interpreted as statistical associations and group-level differences rather than causal effects.

The questionnaire was administered through Google Forms under a standardized administration protocol. It consisted predominantly of closed-ended questions and one optional free-text field. Only closed-ended responses were included in the present quantitative analyses. Free-text responses were not subjected to systematic qualitative coding or thematic analysis; consequently, the study was classified as a quantitative survey rather than a mixed-methods investigation.

Before beginning the questionnaire, respondents viewed an information page explaining the purpose of the study, its approximate duration, the voluntary nature of participation, the intended use and protection of the data, and their right to discontinue before submitting the form. Proceeding to the questionnaire and submitting the completed form constituted electronic informed consent. Questionnaire completion required approximately 15–17 min.

### 2.2 Participants and recruitment

Participants were recruited using non-probability convenience and network-based sampling. The survey link was distributed electronically through educational, professional, community, social-media, and personal networks, and regional contacts additionally circulated the survey outside the largest urban centers to broaden geographic and residential coverage. This procedure facilitated the inclusion of respondents from different settlement types and regions but did not constitute probability sampling and did not yield a nationally representative sample.

Eligibility criteria were:

(a) Age between 18 and 65 years;
(b) Current residence in the Kyrgyz Republic;
(c) Completion of the core standardized measures.

The final analytic sample for the present manuscript was obtained after screening all available responses for exact duplicate records, age eligibility, current residence in the Kyrgyz Republic, and completion of the core standardized measures. This procedure resulted in a final analytic sample of 242 eligible adults. The sample included 192 women (79.3%) and 50 men (20.7%). Participants ranged in age from 18 to 65 years (M = 26.28, SD = 10.74, Mdn = 22). Most participants identified as ethnic Kyrgyz (204; 84.3%). Urban residents comprised 79.8% of the sample (*n* = 193), and 20.2% (*n* = 49) resided in rural settlements. Regarding education, 98 participants (40.5%) had completed higher education, 82 (33.9%) reported incomplete or ongoing higher education, 46 (19.0%) had completed general secondary education, and 16 (6.6%) had vocational or specialized secondary education. Detailed sociodemographic characteristics are presented in Table 1.

**Table 1:** Sociodemographic characteristics of the sample (*N* = 242).

Characteristic	Category	*N*	%
Gender	Women	192	79.3
	Men	50	20.7
Age (years)	M = 26.28, SD = 10.74, Mdn = 22, range 18–65		
Ethnicity	Kyrgyz	204	84.3
	Other	38	15.7
Residence	Urban	193	79.8
	Rural	49	20.2
Education	Completed higher	98	40.5
	Incomplete/ongoing higher	82	33.9
	General secondary	46	19.0
	Vocational/specialized secondary	16	6.6

The high proportion of women may reflect the gender salience of the survey topic, the recruitment channels used, and possible self-selection among respondents interested in family and gender-related issues. Participation was voluntary, and no financial or academic compensation was provided. The questionnaire did not request names, contact details, or other direct identifiers, and the analytic dataset was de-identified. Because the invitation was distributed through multiple online and interpersonal channels, the number of individuals who viewed the invitation without completing the questionnaire could not be established, and an exact response rate could not be calculated. The online procedure may have favored individuals with greater internet access, digital literacy, educational engagement, and interest in gender-related topics. The over-representation of women, urban residents, younger adults, and respondents with current or completed higher education is therefore treated as a constraint on population-level generalization rather than as evidence of national representativeness, and it is addressed in the Limitations (Section 5).

An *a priori* power analysis was impracticable because the online convenience sampling precluded precise estimation of the eligible population size. A sensitivity power analysis conducted in G*Power 3.1 (Faul et al., 2007) indicated that, for a fixed-effects multiple-regression model with seven predictors at *α* = 0.05 and statistical power of 0.80, the analytic sample was adequate to detect a small overall effect (*f*^2^ ≈ 0.06). This calculation characterizes the statistical sensitivity of the available sample and is not an a priori sample-size determination.

### 2.3 Measures

The survey battery comprised an author-designed questionnaire and three established instruments:

1 The 12-item short form of the Ambivalent Sexism Inventory (ASI-12);
2 The 10-item Gender Role Beliefs Scale (GRBS-10)
3 L. G. Pochebut’s Cultural-Value Orientations Questionnaire.

All standardized instruments were presented in a bilingual Kyrgyz–Russian format within the same Google Forms questionnaire: each item and response option appeared in Kyrgyz and Russian side by side. This procedure was intended to increase comprehensibility for respondents in the Kyrgyz Republic. Item wording was prepared using forward translation and back-translation and was reviewed independently by two bilingual psychologists familiar with psychological assessment and the Kyrgyz sociocultural context, with attention to semantic correspondence, comprehensibility, and cultural appropriateness, following established recommendations for cross-cultural instrument adaptation (Beaton et al., 2000). Because respondents were not assigned to separate language versions and the language used to read each item was not recorded, distinct Kyrgyz- and Russian-language groups could not be defined; consequently, configural, metric, or scalar measurement invariance across language versions could not be estimated. The adaptation is therefore presented as evidence of linguistic and content review, not as full psychometric validation or proof of cross-language equivalence. Internal-consistency and exploratory factor-analytic evidence for the present sample are reported below and in Table 2.

**Table 2:** Descriptive statistics and internal consistency of the scale scores (*N* = 242).

Measure	M	SD	Range	*α*	*ω*
Benevolent sexism (0–5)	3.01	1.14	0–5	0.83	0.83
Hostile sexism (0–5)	1.92	1.07	0–5	0.83	0.83
ASI-12 composite (0–5)	2.46	0.94	0–5	0.86	0.86
GRBS Traditionalism Index (1–7)	4.24	1.09	1–7	0.70	0.71

Higher scores indicate stronger endorsement of the respective construct. The CVOQ social-relations Option B indicator is a binary item-level variable (39.3% endorsed), based only on the social-relations item.

#### 2.3.1 Author-designed questionnaire

The author-designed questionnaire comprised 25 items assessing sociodemographic characteristics and stereotypical conceptions of women’s roles in family, occupational, public, and media contexts, including dominant social and media representations of women, perceptions of domestic and caregiving responsibilities, family decision-making, women’s professional participation and autonomy, and the perceived influence of social expectations on women’s opportunities. The module included single-choice, matrix-format, and multiple-response items. For multiple-response items, each alternative was coded as a separate binary variable, and percentages were calculated relative to the number of respondents providing a valid answer; consequently, percentages across alternatives could exceed 100%. Because the questionnaire assessed several conceptually distinct domains using heterogeneous response formats, it was not treated as a unidimensional scale, and a single reliability coefficient was not computed for it. The questionnaire was pilot-tested with a separate group of 30 respondents (not included in the analytic sample) to refine item wording, response alternatives, and cultural comprehensibility.

#### 2.3.2 Ambivalent sexism inventory—short form (ASI-12)

Ambivalent sexism was assessed using the 12-item short form of the Ambivalent Sexism Inventory, based on the original instrument (Glick and Fiske, 1996) and the short-form structure examined by Rollero et al. (2014). The instrument comprises two theoretically related but distinguishable six-item subscales: Hostile Sexism, assessing antagonistic attitudes toward women perceived as challenging established gender relations, and Benevolent Sexism, assessing subjectively positive but paternalistic beliefs that portray women as deserving male protection and as particularly suited to conventional feminine and family roles. The instrument was selected for its capacity to capture the ambivalent character of gender attitudes, in which subordinating expectations may be expressed through ostensibly positive content. Items were rated on a six-point scale from 0 (*strongly disagree*) to 5 (*strongly agree*), and subscale means were computed separately, with higher scores indicating stronger endorsement. The two subscale means served as the principal ASI indicators; an overall ASI-12 mean was computed for descriptive purposes but was not interpreted as evidence of a single underlying dimension. In the present sample, internal consistency was good for the Benevolent Sexism subscale (*α* = 0.83, *ω* = 0.83), the Hostile Sexism subscale (*α* = 0.83, *ω* = 0.83), and the ASI-12 composite (*α* = 0.86, *ω* = 0.86). The dimensional structure of the ASI-12 was additionally examined through exploratory factor analysis (Section 2.4).

#### 2.3.3 Gender role beliefs scale—short version (GRBS-10)

Gender-role beliefs were assessed using the 10-item short version of the Gender Role Beliefs Scale, developed by Brown and Gladstone (2012) from the original instrument (Kerr and Holden, 1996). The measure addresses interpersonal etiquette, women’s employment, domestic responsibilities, family decision-making, occupational roles, and behavioral expectations for women and men, and was selected for its brevity and cross-cultural applicability. Items were rated on a seven-point scale from 1 (*strongly agree*) to 7 (*strongly disagree*). In accordance with the published scoring procedure, the egalitarian-keyed item was reverse-coded before the composite was formed. To align the composite with the study’s focus on traditionalism, scores were oriented—through a linear reflection that preserves the rank order and distribution of respondents—so that higher values indicated stronger endorsement of traditional gender-role beliefs and lower values indicated more egalitarian beliefs; this composite is termed the GRBS Traditionalism Index. In the present sample, internal consistency was acceptable for research purposes (Cronbach’s *α* = 0.70). McDonald’s omega was similar (*ω* = 0.71), consistent with the brevity and content heterogeneity of the short form, which combines restrictive-traditional and benevolent-etiquette content. The composite was retained as a global index of traditionalism following the original scoring procedure (Brown and Gladstone, 2012), and its modest reliability was considered when interpreting associations involving the index. Future validation studies with larger samples should further examine its factor structure.

#### 2.3.4 Cultural-value orientations questionnaire

Cultural-value orientations were assessed using L. G. Pochebut’s Cultural-Value Orientations Questionnaire, as presented in *Cross-Cultural and Ethnic Psychology* (Pochebut, 2012). The questionnaire is grounded in the cross-cultural value-orientation tradition associated with Kluckhohn and Strodtbeck’s framework of value orientations (Kluckhohn and Strodtbeck, 1961). It distinguishes three broad types of cultural orientation: traditional culture (TK), modern culture (SK), and dynamically developing culture (DRK). The instrument comprises five forced-choice items addressing orientation toward time, relationships between people and nature, beliefs about human nature, the organization of social relationships, and preferred forms of activity.

For the present study, the questionnaire was presented in a bilingual Kyrgyz–Russian format within the same Google Forms survey: each item and response option appeared in Kyrgyz and Russian side by side. The Russian wording followed the version of the questionnaire used by Pochebut (2012), and the Kyrgyz wording was prepared through translation and expert review to preserve the intended meaning and ensure comprehensibility for respondents in the Kyrgyz Republic. Because participants were not assigned to separate Kyrgyz- or Russian-language versions and the language primarily used by each respondent was not recorded, measurement invariance across language versions could not be estimated.

Because each item represents a distinct cultural domain rather than an item of a single unidimensional scale, responses were analyzed as categorical indicators, and internal-consistency coefficients were not considered appropriate for this measure. Across the full five-item questionnaire, responses were summarized according to Pochebut’s three culture types. The social-relations item was additionally examined separately because this was the item previously used as an exploratory cultural-value predictor in the regression model. To allow readers to evaluate this item directly, the exact wording of the administered social-relations item and all three response alternatives in Kyrgyz and Russian, together with English translations prepared for reporting purposes, is provided in Supplementary Table 1. The English wording was not administered to participants.

In the analytic sample, 41.7% selected Option A, corresponding to the hierarchical (lineal) ancestry response; 39.3% selected Option B, corresponding to the collectivist (collateral) extended-family response; and 19.0% selected Option C, corresponding to the individualist response. Thus, only within this particular social-relations item did the response of interest represent a collectivist (collateral) relational orientation in Kluckhohn and Strodtbeck’s (1961) framework and should not be treated as an overall or multidimensional measure of cultural orientation. Because this item contains three conceptually distinct response alternatives, analyses involving the social-relations item were conducted using its full three-category structure through dummy coding, with Option C used as the reference category. Responses to this item are interpreted only as exploratory categorical indicators of social-relations orientation rather than as validated multidimensional measures of collectivism, family collectivism, individualism, or related cultural constructs. The study battery did not include an independent multi-item scale of collectivism or related cultural constructs; therefore, external convergent validation of this specific indicator was not available. This limitation is acknowledged in Section 5.

### 2.4 Data preparation and statistical analysis

Analyses were conducted in IBM SPSS Statistics 28, with supplementary internal-consistency and factor analyses in Jamovi 2.3, following established guidance for applied psychological research and regression modeling. The dataset was screened for duplicate records, age eligibility, and completeness; the standardized measures contained no missing responses among the 242 eligible participants, and no imputation was performed. Descriptive statistics comprised frequencies and percentages for categorical variables and means, standard deviations, medians, and observed ranges for continuous composite scores; multiple-response percentages were identified explicitly in the corresponding tables and figure notes. Internal consistency was evaluated using Cronbach’s alpha and McDonald’s omega, interpreted in conjunction with scale length, dimensionality, and item content.

The dimensional structure of the ASI-12 was examined through exploratory factor analysis to assess whether the response structure was broadly consistent with the theoretically expected distinction between hostile and benevolent sexism. Sampling adequacy was evaluated using the Kaiser–Meyer–Olkin measure and Bartlett’s test of sphericity; factors were extracted using principal-axis factoring with oblique rotation (because the two dimensions were expected to correlate), and the number of retained factors was informed by parallel analysis, the scree plot, theoretical interpretability, and the pattern of loadings. This analysis was treated as sample-specific exploratory evidence rather than as a formal validation of the language versions.

The confirmatory hypotheses were evaluated as follows. *H1* (benevolent sexism endorsed more strongly than hostile sexism) was tested with a paired-samples t-test comparing Benevolent and Hostile Sexism, reporting the mean difference, its 95% confidence interval, and Cohen’s *d*_*z*; the distribution of difference scores was inspected beforehand. *H2* (both forms of sexism positively associated with traditionalism) was tested with bivariate correlations. *H3* was examined in two steps: first, through a point-biserial correlation between the GRBS Traditionalism Index and the binary Option B social-relations indicator (coded 1 = Option B, the collectivist (collateral) extended-family response in Pochebut’s social-relations item; 0 = Options A or C); and second, through a robustness check using the full three-category structure of the item. *H4* was tested with a hierarchical multiple linear regression predicting the GRBS Traditionalism Index, in which age, gender, educational attainment, and urban versus rural residence were entered in Block 1 as sociodemographic covariates and Benevolent Sexism, Hostile Sexism, and the Option B social-relations indicator were entered in Block 2 as the theoretically central attitudinal variables and exploratory cultural-value indicator. The incremental contribution of Block 2 was evaluated using the change in *R*^2^ and the corresponding *F*-change test, and results included unstandardized and standardized coefficients, standard errors, 95% confidence intervals, exact *p*-values where applicable, *R*^2^, adjusted *R*^2^, and Δ*R*^2^. Gender was coded 0 = women, 1 = men; residence 0 = rural, 1 = urban; education was treated as an ordered variable (general secondary; vocational/specialized secondary; incomplete/ongoing higher; completed higher). The term predictor denotes a variable’s statistical role in the model and does not imply temporal or causal precedence; no interaction terms were included, so no variable was interpreted as a moderator.

The dominant media representations of women (addressing the study’s descriptive objective) were compared using Cochran’s *Q* test for the overall within-participant comparison of multiple-response endorsements, followed, where the omnibus test was significant, by pairwise McNemar tests with Holm-adjusted significance levels. The exploratory research question concerning demographic correlates (age, gender, education, residence) was examined using the correlations described above and group comparisons: because of the substantial imbalance between women and men, group differences in continuous scores were tested with Welch’s independent-samples *t*-tests, reporting mean differences, 95% confidence intervals, and Hedges’ *g*; associations between categorical variables used Pearson’s *χ*^2^ tests when expected-frequency assumptions were satisfied and Fisher’s exact test otherwise.

Regression assumptions were assessed using residual and scatterplots (linearity, homoscedasticity), *Q*–*Q* plots (residual normality), the Durbin–Watson statistic (independence of residuals), and variance inflation factors and tolerance statistics (multicollinearity); standardized residuals, leverage values, and Cook’s distance were inspected to identify influential observations, and HC3 heteroscedasticity-consistent standard errors were examined as a sensitivity check. Normality of regression residuals was additionally evaluated using the Shapiro–Wilk test; homoscedasticity was assessed through residual plots. All inferential tests were two-sided with *α* = 0.05; exact *p*-values were reported to three decimal places (values below 0.001 as *p* < 0.001), and Holm correction was applied within families of secondary pairwise comparisons. Statistical significance was interpreted together with effect sizes, confidence intervals, and consistency with the theoretical framework. The use of generative artificial intelligence is disclosed separately in the Generative AI Statement and did not form part of the research methodology.

### 2.5 Ethical considerations

At the time the research was conducted, the institution did not maintain a formal institutional research ethics committee or an equivalent independent review body responsible for non-clinical social and behavioral research, and a prospective institutional ethics review and approval number were therefore not available. This institutional circumstance is reported transparently and is not presented as an exemption granted by an ethics committee. The study was non-interventional and comprised a minimal-risk, anonymous online survey; it involved no clinical procedures, experimental interventions, biological materials, medical records, or intentional collection of direct personal identifiers. It was designed and carried out in accordance with the ethical principles of the World Medical Association’s Declaration of Helsinki, including respect for autonomy, voluntary participation, informed consent, minimization of foreseeable risk, protection of privacy and confidentiality, and the right to withdraw. Before accessing the questionnaire, respondents viewed an information page describing the purpose and procedures of the study, its approximate duration, the voluntary nature of participation, the intended use and protection of the data, and their right to discontinue before submitting their responses; proceeding to the questionnaire and submitting the completed form constituted electronic informed consent. Only responses from adults aged 18 years or older were retained, any potentially identifying information inadvertently entered in the optional free-text field was removed before analysis, and findings are reported only in aggregate form.

## 3 Results

### 3.1 Preliminary analyses

The analytic sample comprised 242 complete cases with no missing values on the study variables. Internal-consistency estimates are reported in Table 2. Reliability was good for the ASI-12 composite (*α* = 0.86, *ω* = 0.86) and for both the Benevolent Sexism (*α* = 0.83, *ω* = 0.83) and Hostile Sexism (*α* = 0.83, *ω* = 0.83) subscales, and acceptable for the GRBS Traditionalism Index (*α* = 0.70); McDonald’s omega for the latter was similar (*ω* = 0.71), consistent with the content heterogeneity of the short scale.

The dimensional structure of the ASI-12 was examined through exploratory factor analysis. Sampling adequacy was good (Kaiser–Meyer–Olkin = 0.87), and Bartlett’s test of sphericity was significant, *χ*^2^(66) = 1074.7, *p* < 0.001. Principal-axis factoring with oblique rotation yielded a two-factor solution in which the six benevolent-sexism items and the six hostile-sexism items loaded on separate factors, broadly reproducing the theoretically expected Hostile/Benevolent structure in the present sample.

Parallel analysis based on 1,000 random datasets retained two factors for the ASI-12, which accounted for 27.9 and 27.4% of the variance (cumulative 55.4%) and were moderately correlated (*r* = 0.45); pattern loadings and communalities are reported in Table 3. For the GRBS-10, sampling adequacy was acceptable (Kaiser–Meyer–Olkin = 0.74) and Bartlett’s test of sphericity was significant, *χ*^2^(45) = 611.6, *p* < 0.001; parallel analysis indicated three components consistent with the content breadth of the short scale, and a single general-traditionalism factor accounted for 28.7% of the variance. The composite was therefore retained as the published short-form index and interpreted with corresponding caution. Confirmatory factor analysis and formal measurement-invariance testing were not undertaken, because the present sample size relative to model complexity favored exploratory examination; both remain priorities for future validation in larger, language-stratified samples.

**Table 3:** Exploratory factor analysis of the ASI-12 (*N* = 242).

Item	*F*1 (Hostile)	*F*2 (Benevolent)	*h* ^2^
BS1	0.03	0.64	0.41
HS2	0.48	0.21	0.28
HS3	0.83	−0.07	0.69
BS4	0.07	0.67	0.45
HS5	0.75	−0.01	0.57
HS6	0.66	0.04	0.44
BS7	−0.01	0.81	0.65
BS8	0.09	0.78	0.61
HS9	0.78	−0.03	0.61
HS10	0.88	−0.11	0.78
BS11	−0.17	0.76	0.60
BS12	−0.01	0.74	0.54

*N* = 242. Principal-axis factoring with direct oblimin rotation; primary loadings appear on the theoretically expected factor. *F*1 = Hostile Sexism; *F*2 = Benevolent Sexism; *h*^2^ = communality. Eigenvalues: *F*1 = 4.73, *F*2 = 1.86; the two factors accounted for 27.9 and 27.4% of the variance, respectively (cumulative 55.4%). KMO = 0.87; Bartlett’s *χ*^2^(66) = 1074.7, *p* < 0.001; parallel analysis (1,000 datasets) retained two factors; inter-factor correlation *r* = 0.45. For the GRBS-10 (KMO = 0.74; Bartlett’s *χ*^2^(45) = 611.6, *p* < 0.001), parallel analysis indicated three components consistent with the content breadth of the short scale; a single general-traditionalism factor accounted for 28.7% of the variance, and the composite was retained as the published short-form index.

Regression diagnostics indicated that the assumptions of the multiple-regression model were adequately met. Residuals were approximately normally distributed (Shapiro–Wilk *W* = 0.99, *p* = 0.42), the Durbin–Watson statistic (1.90) indicated independence of residuals, and multicollinearity was negligible (all variance inflation factors ≤1.41; Table 4). Examination of standardized residuals, leverage values, and Cook’s distance revealed no influential observations that materially altered the results, and HC3 heteroscedasticity-consistent estimates did not differ substantively from the conventional estimates.

**Table 4:** Hierarchical regression predicting the GRBS Traditionalism Index (*N* = 242).

Predictor	*B*	SE *B*	*β*	95% CI (*B*)	*p*	VIF
Block 1 (*R^2^* = 0.04, adj. *R^2^* = 0.03)						
Age	0.021	0.007	0.21	[0.01, 0.03]	0.003	—
Male	0.221	0.174	0.08	[−0.12, 0.56]	0.205	—
Education	−0.021	0.067	−0.02	[−0.15, 0.11]	0.753	—
Urban	0.061	0.174	0.02	[−0.28, 0.40]	0.727	—
Block 2 (*R^2^* = 0.27, adj. *R^2^* = 0.25, Δ*R*^2^ = 0.23***)						
Age	0.008	0.006	0.08	[−0.01, 0.02]	0.236	1.31
Male	0.084	0.157	0.03	[−0.22, 0.39]	0.594	1.10
Education	−0.022	0.059	−0.02	[−0.14, 0.09]	0.713	1.17
Urban	0.062	0.156	0.02	[−0.24, 0.37]	0.692	1.06
Benevolent sexism	0.244	0.061	0.26	[0.13, 0.36]	< 0.001	1.29
Hostile sexism	0.305	0.067	0.30	[0.17, 0.44]	< 0.001	1.41
Option B social-relations response	0.278	0.128	0.13	[0.03, 0.53]	0.031	1.06

Dependent variable = GRBS Traditionalism Index. Durbin–Watson = 1.90. Δ*R*^2^ ***p < 0.001.

### 3.2 Sample characteristics

Sociodemographic characteristics of the sample are presented in Table 1. The sample was predominantly female (79.3%), urban (79.8%), and ethnically Kyrgyz (84.3%), with a mean age of 26.28 years (SD = 10.74). These characteristics are consistent with the online, convenience-based recruitment and are considered when interpreting the findings (Section 5).

### 3.3 Perceived media images of women

**Figure 1:**
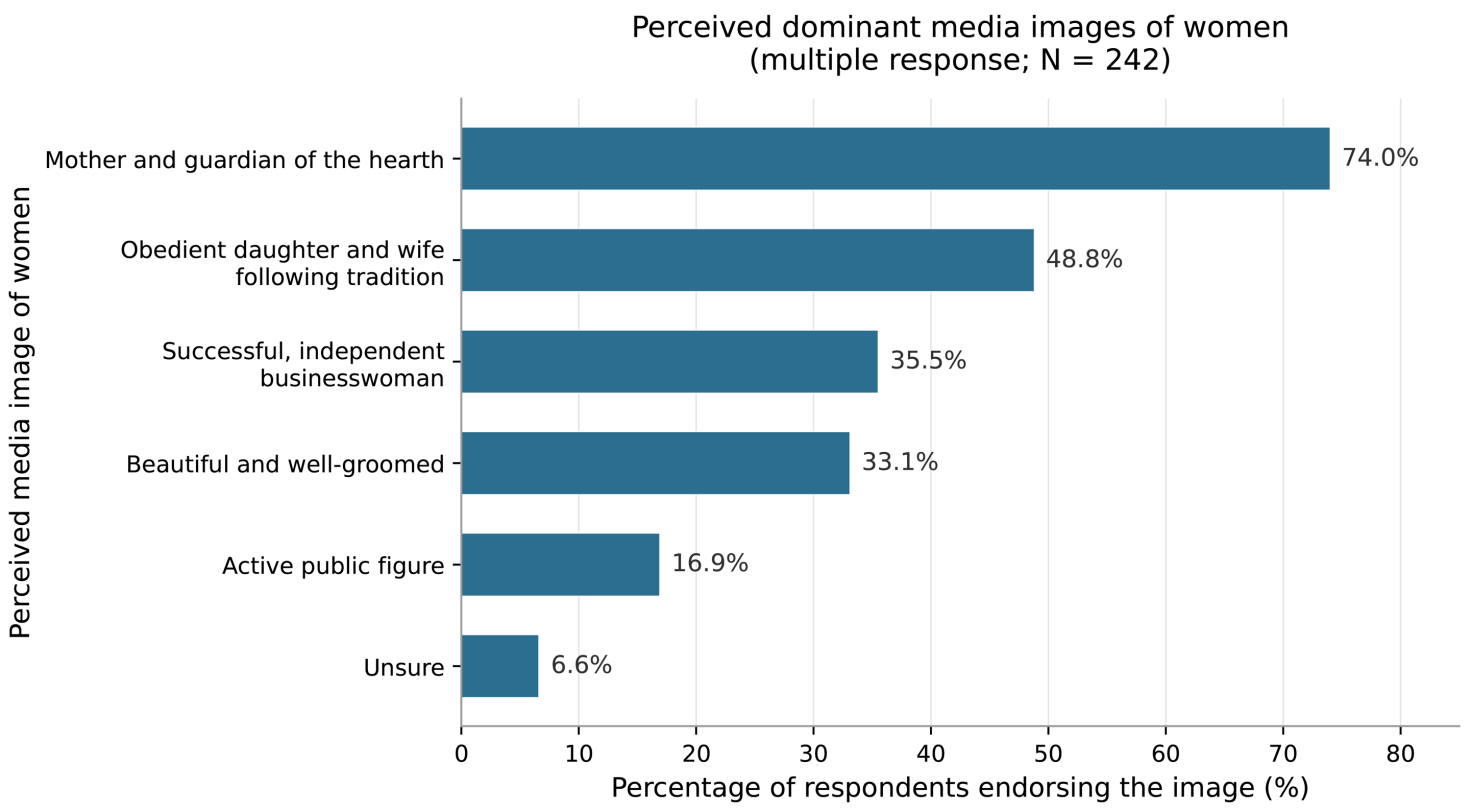
Perceived dominant media images of women (multiple response; *N* = 242).

Respondents indicated which images of women they perceived as most endorsed in the contemporary Kyrgyz media landscape (multiple response; Figure 1). The image of the “mother and guardian of the hearth” was endorsed most frequently (74.0%), followed by “obedient daughter and wife following tradition” (48.8%), “successful and independent businesswoman” (35.5%), “beautiful and well-groomed” (33.1%), and “active public figure” (16.9%); 6.6% were unsure. A Cochran’s *Q* test indicated that endorsement differed significantly across the five image categories, *Q*(4) = 180.3, *p* < 0.001. Follow-up pairwise McNemar comparisons (Holm-adjusted) showed that the “mother and guardian of the hearth” image was endorsed significantly more often than each of the other images (all *p* < 0.001).

### 3.4 Descriptive statistics and ambivalent sexism (*H1*)

Descriptive statistics for the composite scores are reported in Table 2. Consistent with H1, benevolent sexism (*M* = 3.01, *SD* = 1.14) was endorsed substantially more strongly than hostile sexism (*M* = 1.92, *SD* = 1.07); a paired-samples *t*-test confirmed a large difference, *t*(241) = 14.65, *p* < 0.001, *d_z* = 0.94, 95% CI of the mean difference [0.95, 1.24]. The GRBS Traditionalism Index (*M* = 4.24, *SD* = 1.09) was slightly above the scale midpoint of 4, *t*(241) = 3.42, *p* < 0.001, *d* = 0.22, indicating a modest overall lean toward traditional gender-role beliefs. On the Cultural-Value Orientations Questionnaire, responses corresponding to modern culture (SK) predominated across the full five-item measure: 583 of 1,210 item selections (48.2%) were classified as SK, compared with 313 selections (25.9%) for traditional culture (TK) and 314 selections (26.0%) for dynamically developing culture (DRK). On the social-relations item specifically, 39.3% selected Option B, the collectivist (collateral) extended-family response in Pochebut’s social-relations item; 41.7% selected Option A (hierarchical (lineal) orientation), and 19.0% selected Option C (individualist orientation). Although Option B was used analytically as an exploratory item-level social-relations indicator, its administered wording referred to extended family; therefore, it is interpreted here only as an item-level collectivist (collateral) extended-family response rather than as a validated multidimensional measure of collectivism.

### 3.5 Bivariate associations (*H2* and *H3*)

Bivariate correlations among the study variables are reported in Table 5. Consistent with *H2*, gender-role traditionalism was positively associated with both benevolent sexism, *r*(240) = 0.40, *p* < 0.001, 95% CI [0.29, 0.50], and hostile sexism, *r*(240) = 0.44, *p* < 0.001, 95% CI [0.33, 0.54]; the two sexism subscales were themselves moderately correlated (*r* = 0.45, *p* < 0.001). In the primary binary specification of H3, the Option B social-relations indicator was positively, though weakly, associated with traditionalism, *r*(240) = 0.16, *p* = 0.016, 95% CI [0.03, 0.28]. Benevolent sexism was not significantly associated with the Option B indicator (*r* = 0.04, *p* = 0.514). Traditionalism was also positively associated with age (*r* = 0.19, *p* = 0.003) but not with education (*r* = 0.06, *p* = 0.392) or urban residence (*r* = 0.01, *p* = 0.883).

**Table 5:** Intercorrelations among study variables (*N* = 242).

Variable	1	2	3	4	5	6	7	8
1. Traditionalism	—							
2. Benevolent sexism	0.40***	—						
3. Hostile sexism	0.44***	0.45***	—					
4. Option B social-relations response	0.16*	0.04	0.04	—				
5. Education	0.06	0.07	0.10	−0.01	—			
6. Urban residence	0.01	0.10	−0.07	−0.10	0.06	—		
7. Age	0.19**	0.10	0.26***	0.18**	0.37***	0.00	—	
8. Male	0.05	0.00	0.16*	−0.12	−0.02	−0.15*	−0.13*	—

Coefficients are Pearson *r*, point-biserial, or Spearman ρ as appropriate to variable type. Male coded 1 = men, 0 = women; Urban coded 1 = urban, 0 = rural; Option B social-relations indicator coded 1 = Option B on the social-relations item (collectivist (collateral) extended-family response), 0 = Options A or C. *p < 0.05, **p < 0.01, ***p < 0.001.

### 3.6 Hierarchical regression predicting gender-role traditionalism (*H4*)

A hierarchical multiple regression predicted the GRBS Traditionalism Index (Table 4). In Block 1, the sociodemographic covariates accounted for a small proportion of variance, *R^2^* = 0.04, and only age was a significant predictor (*β* = 0.21, *p* = 0.003). Adding the attitudinal variables and the exploratory cultural-value indicator in Block 2 produced a substantial and significant increase in explained variance, Δ*R^2^* = 0.23, *F*-change (3, 234) = 24.28, *p* < 0.001, with the full model accounting for 27.0% of the variance (adjusted *R^2^* = 0.25). Partly consistent with H4, hostile sexism (*β* = 0.30, *p* < 0.001) and benevolent sexism (*β* = 0.26, *p* < 0.001) were the strongest predictors of traditionalism, and the Option B social-relations indicator remained a significant, if modest, statistical predictor in the primary binary model (*β* = 0.13, *p* = 0.031). None of the sociodemographic variables was a significant predictor in the full model; notably, the association of age with traditionalism was no longer significant once ambivalent sexism was included (*β* = 0.08, *p* = 0.236).

### 3.7 Exploratory demographic analyses (research question)

The exploratory analyses examined how demographic characteristics related to the study variables. Welch’s *t*-tests indicated that men reported higher hostile sexism than women (M = 2.25 vs. 1.83), *t* = 2.45, *p* = 0.017, Hedges’ *g* = 0.40, whereas benevolent sexism did not differ by gender (M = 3.01 vs. 3.01, *p* = 0.976) and gender-role traditionalism did not differ significantly between men and women (M = 4.35 vs. 4.21, *p* = 0.484, *g* = 0.13). At the bivariate level, older age was associated with higher hostile sexism (*r* = 0.26, *p* < 0.001) and higher traditionalism (*r* = 0.19, *p* = 0.003); however, as noted above, the age–traditionalism association was accounted for by ambivalent sexism in the multivariate model. Education and urban versus rural residence were not significantly associated with gender-role traditionalism (Table 5). Selection of Option B on the social-relations item was more frequent among older respondents (*r* = 0.18, *p* = 0.006).

In summary, H1 and H2 were clearly supported: benevolent sexism exceeded hostile sexism, and both forms of sexism were positively associated with gender-role traditionalism. H3 and H4 received partial and cautious support. The Option B social-relations indicator showed a weak association with traditionalism and was a modest predictor in the primary binary model, but the robustness check below showed that this association was not robust when the full three-category structure of the item was examined. Across all specifications, benevolent and hostile sexism remained the most consistent predictors of gender-role traditionalism. The exploratory analyses indicated that hostile sexism was more pronounced among men and among older respondents, whereas education and place of residence were not significantly related to traditionalism in this sample.

#### 3.7.1 Robustness check: three-category treatment of the social-relations item

Because the social-relations item comprises three response alternatives, its association with gender-role traditionalism was re-examined using the full categorical structure rather than a binary Option B indicator. Descriptively, mean traditionalism was highest among respondents selecting Option B, the collectivist (collateral) extended-family response (M = 4.45, SD = 1.08, *n* = 95), intermediate among those selecting Option C, the individualist response (M = 4.21, SD = 1.09, *n* = 46), and lowest among those selecting Option A, the hierarchical (lineal) response (M = 4.06, SD = 1.07, *n* = 101).

The item was then entered into the hierarchical regression as two dummy-coded contrasts, with Option C used as the reference category, alongside age, gender, education, residence, benevolent sexism, and hostile sexism. The three-category social-relations item did not account for significant incremental variance in gender-role traditionalism, Δ*R^2^* = 0.015, *F*(2, 233) = 2.39, *p* = 0.093.

Neither contrast against the Option C reference category reached statistical significance: Option B versus Option C, *B* = 0.235, SE = 0.172, *β* = 0.11, 95% CI [−0.10, 0.57], *p* = 0.174; Option A versus Option C, *B* = −0.063, SE = 0.172, *β* = −0.03, 95% CI [−0.40, 0.27], *p* = 0.713. Variance inflation factors were acceptable (VIFs = 1.91 and 1.94).

The only significant pairwise difference was between Option B and Option A: respondents selecting the collectivist (collateral) extended-family response showed higher gender-role traditionalism than those selecting the hierarchical (lineal) ancestry response *B* = 0.298, SE = 0.140, *β* = 0.13, 95% CI [0.02, 0.57], *p* = 0.035; unadjusted *t*(194) = 2.55, *p* = 0.011. Benevolent sexism (*β* = 0.26) and hostile sexism (*β* = 0.30) remained significant and essentially unchanged across model specifications, both *p* < 0.001.

Thus, the association between the social-relations item and gender-role traditionalism was weak and should be interpreted cautiously. The pattern was mainly driven by the contrast between Option B and Option A and did not constitute robust evidence for an independent contribution of a multidimensional cultural-value orientation. In the terminology of Pochebut’s questionnaire, the result refers only to the collectivist (collateral) relational orientation on this specific social-relations item and should not be generalized to the full five-item cultural-value profile or interpreted as a validated collectivism construct.

## 4 Discussion

The present study provided a quantitative, social-psychological examination of stereotypical conceptions of women’s roles among adults surveyed in the Kyrgyz Republic, integrating ambivalent sexism, gender-role beliefs, cultural-value orientation, and perceived media images within a single design. *H1* and *H2* were clearly supported, whereas *H3* and *H4* received partial and cautious support because the cultural-value indicator was weak and coding-sensitive. Interpretation is framed in associational terms; the cross-sectional design does not permit causal inference, and all conclusions are bounded by the characteristics of the participating sample.

### 4.1 Predominance of benevolent over hostile sexism

The clearest finding was the marked predominance of benevolent over hostile sexism (*H1*), a difference of large magnitude (*d_z* = 0.94). Within Ambivalent Sexism Theory, this pattern is interpretable as the greater social acceptability of subjectively positive, protective attitudes relative to overtly antagonistic ones (Glick and Fiske, 1996; Bareket and Fiske, 2023). In a cultural setting in which protection, family cohesion, and respect for women’s family roles are positively valued and are encoded in constructs such as *Uyat* and *Namys*, benevolent-sexist content may be expressed and endorsed as care or courtesy rather than as prejudice. The comparatively low endorsement of hostile sexism, together with the high endorsement of benevolent sexism, is consistent with the theoretical account in which traditional gender-role arrangements are sustained less through open antagonism than through subjectively positive, protective idealization of family-centered roles. This finding also extends the empirical literature on ambivalent sexism, which has rarely assessed the relative prominence of its two components in post-Soviet Central Asian samples using standardized psychometric measures.

### 4.2 Ambivalent sexism and gender-role traditionalism

Both benevolent and hostile sexism were positively associated with gender-role traditionalism (*H2*), and in the primary binary model, together with the item-level social-relations indicator, they predicted traditionalism beyond sociodemographic characteristics, accounting for a substantial increment in explained variance (*H4*; Δ*R*^2^ = 0.23). In the full model, hostile and benevolent sexism were the strongest predictors. This pattern is consistent with the theoretical claim that hostile and benevolent sexism are associated in a pattern consistent with complementary components of a traditional gender ideology rather than with opposing attitudes: the former is theorized to sanction departures from conventional roles, whereas the latter is theorized to reward conformity to them (Glick and Fiske, 2012; Bareket and Fiske, 2023). That both components independently predicted traditionalism suggests that, in this sample, endorsement of traditional gender-role beliefs was linked not only to antagonism toward role-nonconforming women but also to ostensibly benevolent beliefs about women’s protection and family centrality. The moderate correlation between the two subscales (*r* = 0.45) is likewise consistent with their theorized status as related but distinguishable dimensions.

### 4.3 Cultural orientation and demographic correlates

Across the five-item Cultural-Value Orientations Questionnaire, responses corresponding to modern culture (SK) predominated descriptively. This does not mean that respondents were uniformly “modern” in all domains; rather, it indicates that, across the five forced-choice cultural-value items, SK responses were selected more often than traditional-culture (TK) or dynamically developing-culture (DRK) responses. In Pochebut’s typology, the label modern culture (SK) should not be read as a direct indicator of gender-role egalitarianism or Western-style individualism. It is a typological scoring category that includes, among other features, role-regulated social relations and the coordination of individual decisions with relevant groups, family, and work collectives. On the social-relations item specifically, the response used in the primary binary specification was Option B, which corresponds to the extended-family response, scored as a collectivist (collateral) relational orientation (Kluckhohn and Strodtbeck, 1961). This Option B response was positively, though weakly, associated with gender-role traditionalism in the primary binary specification (H3). However, this item-level result should not be interpreted as evidence that collectivism or any multidimensional cultural construct predicts traditionalism (Tajfel and Turner, 1986; Jost and Kay, 2005). Benevolent sexism was unrelated to the Option B indicator, suggesting that benevolent sexism functioned as a distinct attitudinal construct rather than as a simple reflection of the measured cultural-value response.

Moreover, the robustness check using the full three-category structure of the social-relations item indicated that this item did not explain significant incremental variance in gender-role traditionalism beyond the demographic and sexism-related predictors (Section 3.7.1). The association observed in the binary specification should therefore be interpreted as weak, coding-sensitive, and provisional rather than as evidence for a robust independent effect of cultural orientation. Importantly, this qualification does not alter the study’s central findings: benevolent and hostile sexism remained the most consistent predictors of gender-role traditionalism across model specifications.

Contrary to expectations derived from modernization accounts, neither educational attainment nor urban residence was significantly associated with gender-role traditionalism. Traditional beliefs were thus not confined to less-educated or rural respondents but were distributed across educational and residential groups. This pattern is consistent with Gender Schema Theory, according to which internalized gender schemas can be cognitively economical and resistant to disconfirmation, so that expanded educational and urban participation need not translate directly into more egalitarian beliefs (Bem, 1981). It is also consistent with comparative evidence that gender-role stereotypes remain pronounced in several Central Asian settings across social strata (Yerimpasheva et al., 2023). The null associations should nonetheless be interpreted cautiously given the restricted range of the present sample, which was predominantly urban and highly educated (Section 5).

The exploratory analyses indicated that hostile sexism was more pronounced among men (Hedges’ *g* = 0.40) and increased with age, whereas benevolent sexism and overall traditionalism did not differ significantly by gender. The bivariate association between age and traditionalism was no longer significant once ambivalent sexism was included in the model, suggesting that the somewhat greater traditionalism of older respondents in this sample was statistically accounted for by their higher levels of ambivalent sexism. The absence of a gender difference in benevolent sexism is consistent with prior findings that benevolent sexism is often endorsed comparably by women and men, in line with system-justifying accounts in which complementary, protective beliefs can be adopted by members of the very group they characterize (Jost and Kay, 2005).

### 4.4 Perceived media images and the coexistence of role models

The perceived media landscape was dominated by the family-centered image of the “mother and guardian of the hearth” (74.0%), which was endorsed significantly more often than any other image, followed by the “obedient daughter and wife following tradition” (48.8%). Professional and public images—the “successful businesswoman” (35.5%) and the “active public figure” (16.9%)—were perceived as considerably less prominent. Within Social Role Theory, the salience of family-centered representations is consistent with the observed concentration of women in caregiving and domestic roles giving rise to correspondingly communal, family-oriented expectations (Eagly and Wood, 2012). At the same time, the simultaneous presence of professional and family-centered images is consistent with the coexistence of several culturally available models of womanhood rather than the replacement of traditional images by contemporary ones. The prominence of these perceived representations does not indicate participants’ personal endorsement of them; it reflects their perception of prevailing cultural imagery.

### 4.5 Role strain as a theoretical interpretation

The coexistence of strongly endorsed family-centered expectations with expanding professional participation constitutes a configuration theoretically consistent with role strain (Goode, 1960) and work–family conflict (Netemeyer et al., 1996). It is important to state clearly, however, that the present study did not measure role strain, work–family conflict, cognitive dissonance, self-efficacy, or psychological well-being. The observed pattern therefore describes a structural and attitudinal configuration under which role strain may plausibly arise; it does not establish that participants experienced role conflict or any associated distress. Claims about the psychological experience of competing role demands would require dedicated instruments and, ideally, longitudinal or qualitative evidence. This interpretation is offered as a theoretically motivated direction for future research rather than as a finding of the present study.

### 4.6 Practical implications

Several tentative applied implications follow from the observed associations, subject to the study’s limitations. These implications are oriented toward psychological literacy, informed decision-making, constructive family communication, and family well-being. They are not intended to devalue family roles, motherhood, or culturally significant traditions, and they do not prescribe any particular model of family life.

In education, the finding that gender-role traditionalism was associated with both benevolent and hostile attitudinal content suggests that psychology and social-studies curricula in secondary and tertiary education could address different forms of gender-role beliefs, including openly negative attitudes and protective-paternalistic beliefs that may accompany traditional role expectations. Such discussions could help students reflect on how family expectations, education, and future occupational choices are connected in everyday decision-making.

In university settings, brief discussion-based seminars involving women and men together may be more constructive than confrontational formats. This approach is also consistent with the comparable benevolent-sexism scores observed among women and men in the present sample. Such seminars could focus on role expectations, family communication, educational planning, and psychological adaptation during young adulthood, and their effectiveness could be evaluated with pre–post measures.

In the media sphere, media-literacy activities could invite audiences to reflect on the range of women’s roles represented on television and social media and to consider the breadth of family, educational, professional, and community roles that women occupy in the Kyrgyz Republic. Importantly, the culturally meaningful image of the mother and guardian of the hearth should not be devalued; rather, media and educational materials may present family, educational, professional, and public roles as potentially complementary rather than competing.

In community settings, involving locally respected figures, including educators, elders, psychologists, parents, and community leaders, may make discussions of family expectations and life planning more acceptable than externally framed programs. In the Kyrgyz context, practical work is likely to be more constructive when it is expressed through culturally significant values such as respect, responsibility, family well-being, intergenerational support, patience, honor, and mutual assistance.

For public and institutional programs, initiatives related to education, employment, family support, and youth development may be more workable when framed in terms compatible with family-centered and relational values. Rather than relying on models that do not take local cultural meanings into account, such programs should consider how family responsibility, personal dignity, education, and professional competence can coexist within contemporary Kyrgyz society.

Finally, the results underscore the value of culturally adapted measurement, because constructs such as protection, family responsibility, honor, and respect may carry context-specific meanings. These suggestions are offered as hypotheses for appropriately designed evaluation research rather than as prescriptions, and none follows causally from the present cross-sectional data.

## 5 Limitations and future directions

Several limitations qualify the interpretation of these findings. First, the sample was recruited through non-probability convenience and network-based online sampling and included a higher proportion of women, urban residents, younger adults, and respondents with current or completed higher education. This demographic composition should be considered when interpreting the results. Accordingly, the findings should be generalized cautiously and should not be treated as population-level estimates for the Kyrgyz Republic. The null associations for education and residence should also be interpreted with caution, because the relatively high educational attainment and urban concentration of the sample may have restricted variability in these variables. Because recruitment occurred through multiple online and interpersonal channels, an exact response rate could not be determined, and self-selection bias cannot be excluded.

Second, the study used a cross-sectional design. Therefore, the observed associations cannot support causal or temporal inferences. The direction of the relationships among ambivalent gender attitudes, cultural-value orientation, and gender-role traditionalism cannot be established from these data.

Third, several measurement limitations should be noted. The GRBS Traditionalism Index showed acceptable but modest reliability (*α* = 0.70, *ω* = 0.71), consistent with the brevity and content heterogeneity of the short scale; therefore, associations involving this index should be interpreted with appropriate caution. The social-relations variable derived from Pochebut’s questionnaire was based on a single forced-choice item and provided only a coarse categorical indicator; it should not be interpreted as a validated measure of collectivism, family collectivism, individualism, or related cultural constructs. In addition, the Kyrgyz and Russian versions of the standardized instruments were administered within a single questionnaire rather than through randomly assigned language-version groups. Measurement invariance across language versions could therefore not be tested. The adaptation is therefore presented as evidence of linguistic and content review, not as full psychometric validation or proof of cross-language equivalence. All measures were self-reported and may be subject to social-desirability and common-method biases.

A further limitation concerns the Cultural-Value Orientations Questionnaire. Although the questionnaire is theoretically grounded in the value-orientation tradition and the overall five-item pattern indicated a predominance of modern-culture (SK) responses in the present sample, the regression analysis relied on a single forced-choice social-relations item and therefore could not establish collectivism, family collectivism, individualism, or related cultural constructs as multidimensional variables with adequate reliability or construct validity. This limitation applies regardless of whether the item is treated as a binary indicator or through its full three-category structure. Consistent with this caution, the robustness check showed that the social-relations item did not explain significant incremental variance in gender-role traditionalism when analysed using its full three-category structure (Section 3.7.1). In addition, cross-cultural evidence using comparable value-orientation items suggests that central or moderate alternatives may sometimes be disproportionately selected across culturally diverse groups (Dugarova et al., 2017), raising the possibility that such alternatives can partly function as default or least-objectionable responses rather than as clearly differentiated indicators of the intended construct. In the present sample, however, Option B on the social-relations item was not the most frequently endorsed response (39.3%, compared with 41.7% for Option A), which does not suggest strong over-selection of a single default response for this item. Nevertheless, possible response-style influences cannot be excluded. Future research should therefore employ established multi-item measures of collectivism, family collectivism, and related cultural orientations to validate and extend these findings.

Fourth, several psychological constructs discussed as part of the theoretical interpretation were not directly measured. These include role strain, work–family conflict, self-efficacy, and psychological well-being. The present study therefore cannot directly assess individuals’ actual experience of competing role demands or their psychological consequences. These constructs remain important topics for future research using dedicated instruments.

Fifth, although the analytic sample was adequate to detect small-to-moderate overall regression effects, the study had limited sensitivity to very small effects. Small associations, including the weak association between the Option B social-relations response and gender-role traditionalism, should therefore be interpreted cautiously.

Future research would benefit from probability-based or quota sampling with broader representation of men, rural residents, older adults, and respondents with lower educational attainment. Longitudinal or experimental designs would help clarify the direction of associations among cultural-value orientations, ambivalent gender attitudes, and gender-role traditionalism. Future studies should also use multi-item measures of cultural-value orientations, conduct formal tests of measurement invariance across Kyrgyz- and Russian-language versions, and include direct measures of role strain, work–family conflict, self-efficacy, and psychological well-being. Further research may also examine how role expectations vary across age, education, residence, ethnicity, religiosity, marital status, parental status, and migration experience, thereby providing a more differentiated understanding of the diversity of social and family-role expectations within the Kyrgyz Republic.

## 6 Conclusion

The present study examined women’s role representations, ambivalent gender attitudes, gender-role traditionalism, and cultural-value orientations among adults residing in the Kyrgyz Republic. Within the surveyed sample, family-centered images of women remained especially salient: the most frequently perceived media representation was the image of the mother and guardian of the hearth, followed by the image of an obedient daughter and wife who follows tradition. At the same time, professional and public-role images were also present, indicating that traditional and contemporary representations of women may coexist rather than simply replace one another.

The attitudinal findings showed that benevolent sexism was endorsed substantially more strongly than hostile sexism. This pattern suggests that gender-role traditionalism in the present sample was less closely connected with overtly antagonistic attitudes and more closely associated with subjectively positive, protective-paternalistic beliefs. Both benevolent and hostile sexism were positively associated with gender-role traditionalism and remained significant statistical predictors after adjustment for age, gender, education, and place of residence. By contrast, education and urban residence were not significant predictors in the full regression model, and the social-relations item from the Cultural-Value Orientations Questionnaire showed only weak and coding-sensitive evidence of an independent association with traditionalism. Descriptively, the overall five-item Pochebut profile in this sample was characterized by a predominance of modern-culture (SK) responses, whereas the single social-relations item should be interpreted only as an exploratory categorical indicator. These findings suggest that, in this sample, ambivalent gender attitudes were more proximal correlates of traditional gender-role beliefs than the measured sociodemographic characteristics or the single cultural-value item used in the regression model.

Interpreted within Social Role Theory, Ambivalent Sexism Theory, and complementary cognitive and cultural perspectives, the results indicate that traditional role expectations may be maintained not only through openly negative attitudes but also through culturally meaningful ideals of protection, care, family responsibility, and respect. This interpretation should not be understood as a rejection of family-centered values, motherhood, or culturally significant traditions. Rather, the findings point to the psychological complexity of role expectations in a context where family, education, work, and public participation are all relevant to contemporary social life.

The study contributes quantitative evidence on gender-role beliefs and ambivalent gender attitudes in a post-Soviet Central Asian context where empirical psychological research remains comparatively limited. Its contribution lies in documenting the coexistence of family-centered and contemporary role representations, demonstrating the relevance of benevolent and hostile attitudinal dimensions for gender-role traditionalism, and highlighting the need for culturally grounded measurement and interpretation. Because the study was cross-sectional and based on a non-probability online sample, the findings should be interpreted cautiously and should not be treated as population-level estimates. Future research using more diverse and representative samples, longitudinal designs, and direct measures of role strain, family communication, and psychological well-being would further clarify how role expectations are understood and negotiated within different social groups in the Kyrgyz Republic.

## Data Availability

The datasets presented in this article are not readily available because the study involved anonymous human-participant survey data and participants did not provide explicit consent for public data sharing. Requests to access the datasets should be directed to kamilla.turdubaeva@knu.kg.
