# Supplementary material for: Stereotypical conceptions of women’s roles in contemporary Kyrgyz society: a psychological analysis
Source: Front Psychol. 2026 Aug 5;17:1857963. doi: 10.3389/fpsyg.2026.1857963 (PMC13485722; doi:10.3389/fpsyg.2026.1857963)
Supplement: Supplementary file 1 [file Table_1.pdf]

Supplementary Table S1

Component	Kyrgyz wording administered	Russian wording administered	English translation for reporting
Item stem	Өз ара мамилелер	Взаимоотношения	Relationships
Option A - hierarchical (lineal) orientation: ancestry and origin	Менин маданиятымда адамдар өз ара мамилелердин эң негизги негизи катары мурас менен тегин эсептешет.	В моей культуре люди считают наследство и происхождение самым основным во взаимоотношениях между людьми.	In my culture, people consider heritage and lineage to be the most important basis of relationships between people.
Option B – collectivist (collateral) orientation: extended family	Менин маданиятымда адамдар өз ара мамилелердин эң негизги негизи катары чоң үй-бүлөнү эсептешет.	В моей культуре люди считают большую семью самым основным в своих взаимоотношениях.	In my culture, people consider the extended family to be the most important basis of their relationships.
Option C - individualist orientation: individuality and uniqueness	Менин маданиятымда адамдар өз ара мамилелердин эң негизги негизи катары инсандын индивидуалдуулугун жана өзгөчөлүгүн эсептешет.	В моей культуре люди считают индивидуальность, самобытность личности самым основным в своих взаимоотношениях.	In my culture, people consider individuality and personal uniqueness to be the most important basis of their relationships.

Note. The item was taken from L. G. Pochebut's Cultural-Value Orientations Questionnaire (Pochebut, 2012), which is based on the value-orientation framework of Kluckhohn and Strodtbeck. The questionnaire was administered in a bilingual Kyrgyz–Russian format; the English wording is a translation prepared for reporting purposes and was not administered to participants. In Pochebut's (2012) overall culture-type scoring, Option A, Option B, and Option C contribute to the traditional (TK), modern (SK), and dynamically developing (DRK) profiles, respectively. For the item-level analysis reported here, the three alternatives on this relational dimension were scored by relational orientation following Kluckhohn and Strodtbeck (1961): ancestry and origin (Option A) as hierarchical (lineal), the extended family (Option B) as collectivist (collateral), and individuality (Option C) as individualist. Only the social-relations item is reproduced because this item was used in the exploratory analysis of social-relations orientation. The table reports the administered wording of this item and its correspondence to Pochebut's typological interpretation. Because the regression analysis used only this single forced-choice item, Option B is interpreted as an exploratory item-level collectivist (collateral) extended-family social-relations response rather than as a validated multidimensional measure of collectivism, family collectivism, or cultural orientation.
